# Comparative evaluation of Hand, Rotary and Reciprocation motion on Dentin thickness and instrumentation time in Primary anterior teeth using CBCT: An observational study

**DOI:** 10.4317/jced.60347

**Published:** 2023-05-01

**Authors:** Shashank Kumar, Sathyajith Naik N., Pallavi Vashisth, Shivangi Sharma, Sonal Singh, Deepshikha

**Affiliations:** 1 BDS, MDS, Sr Lecturer, Department of Pedodontics, Institute of Dental Sciences, Bareilly, Uttar Pradesh, India; 2 BDS, MDS, Principal and Head of Department. Professor Dept of Pedodontics. Institute of Dental Sciences. Bareilly. Uttar Pradesh, India; 3 BDS, MDS. Professor. Dept of Pedodontics. Institute of Dental Sciences. Bareilly. Uttar Pradesh, India; 4 BDS, MDS. Reader. Dept of Pedodontics. Institute of Dental Sciences. Bareilly.Uttar Pradesh, India; 5 BDS, MDS. Sr Lecturer. Dept pf Prosthodontics. TMU, Moradabad, U.P; 6 BDS, MDS. Sr Lecturer. Dept of Pedodontics. TMU, Moradabad, U.P

## Abstract

**Background:**

Ultimate goal of BMP is to extirpate the pulp tissue completely, microorganisms, debris & shaping the canal which preserves the original course of the canal to receive an obturating material. Due to various morphological challenges present in deciduous root canal, there is high demand of an improved quality & design of file system with less working length to prevent undesirable complication & reduce treatment time. Aim: To evaluate & inter-compare the dentin thickness and instrumentation time in root dentin of deciduous teeth after BMP in Hand, Rotary & Reciprocation motion with single-file systems.

**Material and Methods:**

60 extracted primary single rooted teeth with un-resorbed roots were included in the study. Teeth were divided into three groups consisting of 20 teeth in each group. In Group-1 Root canal preparation was done with pediatric Hand files, In Group-2 with pediatric Single-file system in rotary motion and in Group-3 with pediatric Single-file system in reciprocating motion. Teeth were scanned before & after preparation with CBCT. Segments were analyzed for dentin thickness at 3mm,5mm and 7mm respectively. Instrumentation time was recorded by an assistant.

**Results:**

Mean instrumentation time of Rotary was least as compared to Reciprocation & Hand respectively, instrumentation time taken by hand filing was significantly higher. Reciprocating filing helps in better dentin debridement at apical and the middle third and no difference was found at the coronal third among all three groups.

**Conclusions:**

Reciprocating filing helps in better dentin debridement and rotary instrumentation requires least time for canal preparation.

** Key words:**Hand Files, Rotary Files, Reciprocating motion, CBCT.

## Introduction

Ultimate goal of Biomechanical Preparation (BMP) is to extirpate the pulp tissue completely, microorganisms, debris & shaping the canal which preserves the original course of the canal to receive an obturating material. Due to various morphological challenges present in deciduous root canal, there is high demand of an improved quality & design of file system with less working length to prevent undesirable complication & reduce treatment time (1). Earlier there was extensive use of Stainless Steel(SS) hand files which has propensity to create aberrations formed by inherent stiffness of stainless steel which limits off its usage in curved narrow canals thereby, hindering obturation (2).

To encounter the drawbacks of SS instruments, many manufacture brought various NiTi Hand & Rotary files system into existence with superior resistance to torsion fracture & enhanced flexibility. Rotary instrumentation brought a quantum leap in the field of endodontics (2) & has proven best in many aspects with only one complication. After various modification of these files through metallurgy they are more fracture resistant to limited number of cases. Therefore, practitioners, with each passing day are exploring the ease of the rotary endodontics in modern-day practice (3). However, it has been abundantly constrained to permanent teeth. These constrains lead to the origination of rotary endodontics in pedodontics. The primary root canal morphology & thinner root dentin restrained the use of these rotary systems in deciduous teeth. To conquer such obstruction, various improved protocols have been put forward to prevent any undesirable complications (4).

The risk of procedural errors is minimized by using Ni-Ti files which follow the original morphology of curved canals in deciduous teeth (5). Rotary filing is faster in deciduous teeth as compared to permanent, due to smaller root canal length (6). With changing trends, much consideration has been aimed towards making pulpectomy a less laborious & more efficient procedure (1).

In the past few years, the introduction of single file system in both manual hand & rotary motion has been in the new concept for deciduous teeth which has transfigured the pulpectomy by making pediatric patient more co-operative as they cause less or no pain during the procedure, reducing operator fatigue, the time needed for canal preparation & minimizing the procedural inaccuracy as compared to sequential files system instruments.

The recently progressed reciprocating movement is asserted to relieve stress on the file by special counter-clockwise (cutting action) & clock-wise (release of instrument) movements & is assumed that the risk of cyclic fatigue of the file caused by tension & compression is reduced by this reciprocating movement (7).

There are few files system which are especially designed for pediatric patients which are single file system. Since, there is limited data available in literature regarding the newly introduced pediatric endodontics files, this study has been planned to evaluate & compare the dentinal microcracks, dentinal thickness and instrumentation time between Hand, Rotary & Reciprocating files instrumentation using CBCT analysis 

The present study was conducted in Department of Pediatric & Preventive Dentistry, I.D.S, Bareilly, Uttar Pradesh, India. 60 extracted deciduous anterior teeth were selected.

## Intervention / Procedure

-Storage: All the selected sample teeth were cleaned of debris & soft tissue remnants, and stored in saline until used and divided into three groups consisting of 20 teeth in each group.

**Figure 1:**
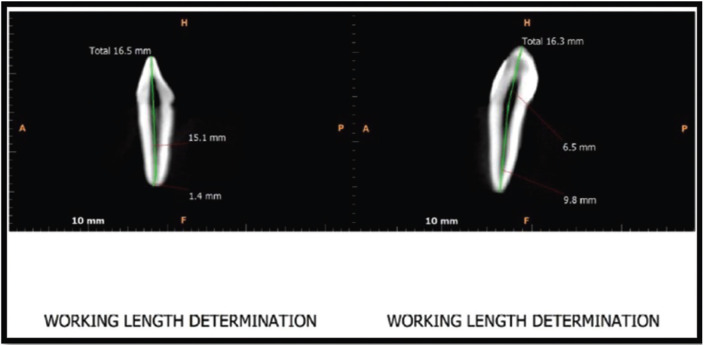
Teeth length measured with the help of CBCT.

**Figure 2:**
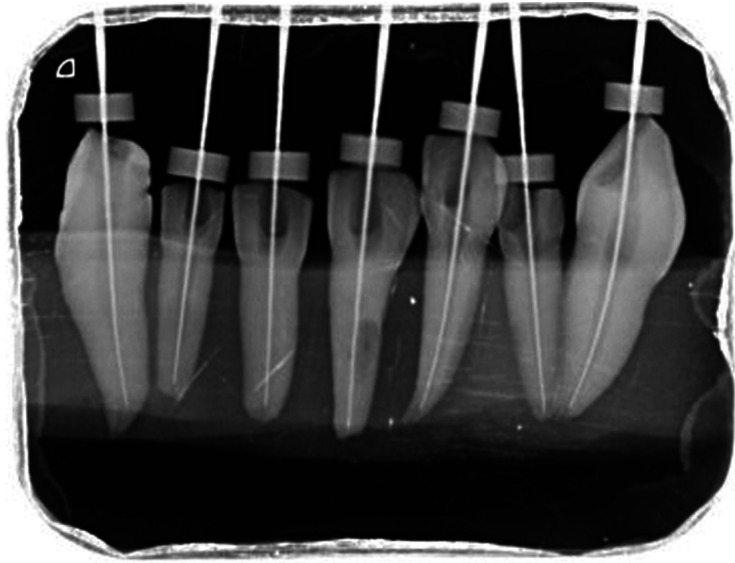
Working length confirmation in RVG.

-Procedure: All the teeth were mounted in acrylic moulds vertically and kept in distilled water to prevent the specimens from drying The tooth length was determined by the pre-procedural CBCT (Fig. 1). The Access opening was done and apical patency were determined by using no. 15 size SS Hand K-file (Dentsply Maillefer) into the root canal and the W/L was set 1mm short of its measurement & confirmed by RVG (VISTA SCAN by DURR DENTAL (Fig. 2). The preparation of canal was done according to the group:

Group -1 (N=20) Kedo-SH manual files (Reeganz Dental Care Pvt. Ltd. India) was used.

Extirpation of the pulp was done with a 2% taper no. 35 size (green) SS H-FILE. After pouring a few drops of EDTA, hand filing was done with U1 (Black color-coded with tip diameter 0.40 Kedo-SH) file with VV taper. After 3-4 strokes chip space was cleaned to prevent file engagement in canal. Irrigation was done with normal saline & hypo. Again EDTA was poured in the canal space & hand filing was done till the file reaches to the WL followed by irrigation.

Group-2 (N=20) Kedo- S+ (Reeganz Dental Care Pvt. Ltd. India) was used in rotary motion.

After pouring a few drops of EDTA, filing was done in brushing motion using A1+ (black color-coded with tip diameter 0.40) file in rotary motion at speed 300rpm & torque 2.2 with X-SMART PLUS Endo motor. Single Stroke Clean (Accor. to Allen Ali Nasseh)45 was done to remove the debris collected in the chip space of file to avoid file engagement. Filing was done till file reaches the W/L followed by irrigation.

Group-3 (N=20) Kedo- S+ (Reeganz Dental Care Pvt. Ltd. India) was used in reciprocation motion.

After pouring a few drops of EDTA, filing was done in pecking motion using A1+ (Black color-coded with tip size 0.40) file in reciprocating motion at pre-set program of RECIPROC ALL with X-SMART PLUS Endo motor. Irrigation was done after every 4-5 strokes to avoid collection of debris within the canal space. Filing was done till file reaches the W/L followed by irrigation.

Examination of roots: Teeth were scanned before & after canal preparation with CBCT. Segments were analyzed at coronal, middle & apical third.

**Figure 3:**
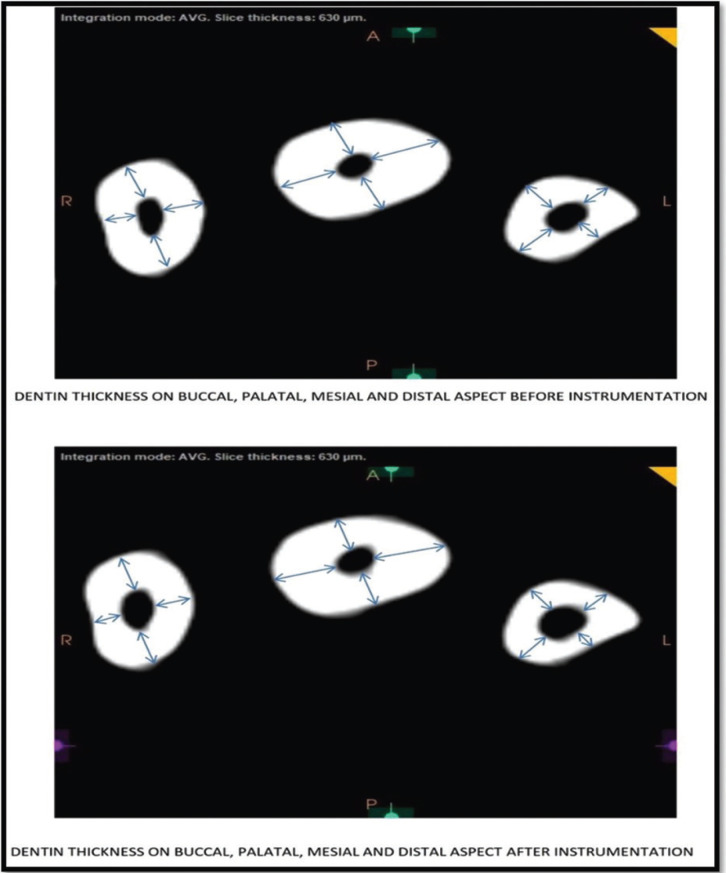
Pre & Post scanned images measuring dentine thickness.

-Assessment of dentine thickness:

The dentine thickness was measured & compared in millimeter from pre to post instrumentation on all four walls i.e labial, palatal, mesial & distal at 3mm, 5mm & 7mm from the root apex (Fig. 3).

-Assessment of instrumentation time:

During filing, the time was recorded using stopwatch. The duration of irrigation & cleaning the file between the instrumentation was not calculated. Therefore, the active filing time was recorded.

## Outcome measures

A. Instrumentation time

The instrumentation time of Group 1, Group 2 and Group 3 ranged between 4.21-9.57, 1.16-4.24 and 1.49-4.17 min respectively with mean (± SD) 6.15 ± 1.70, 2.19 ± 0.90 and 2.96 ± 0.76 min respectively and median 5.44, 2.13 and 3.18 min respectively. The mean instrumentation time of Group 2 was the minimum followed by Group 3 and Group 1, the maximum (Group 3 < Group 2 < Group 1) (Table 1).

**Table 1:**
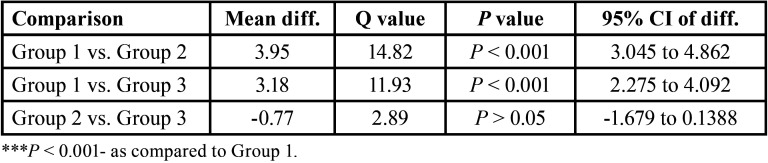
Comparison (*P* value) of difference in mean instrumentation time (min) between groups by Tukey test.

B. Dentin thickness

I. At 3 mm length

The pre and post dentin thickness of three groups at 3 mm length in different sites (buccal, palatal, mesial and distal) is summarised in Table 2. For each site, comparing the difference in pre to post mean change in dentin thickness between groups (i.e. inter groups), Tukey test also showed insignificant (*P* > 0.05) difference in pre to post mean change in dentin thickness between all groups at all sites i.e. did not differ significantly (Table 3).

**Table 2:**
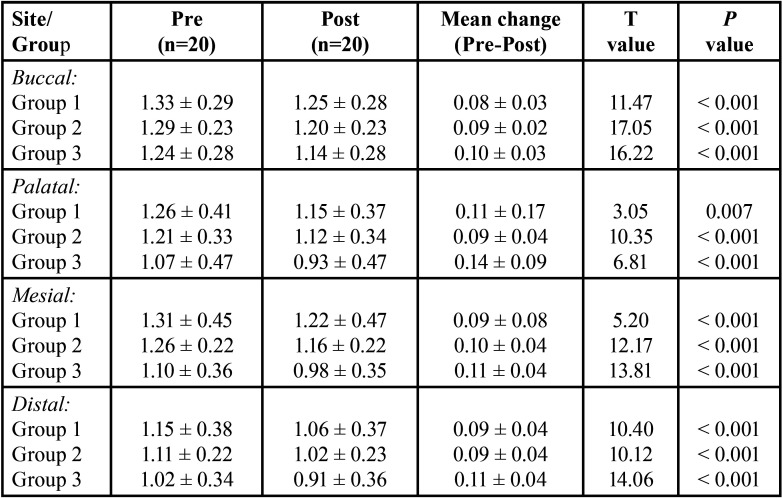
Pre and post dentine thickness (mm) of three groups at 3 mm length in different sites.

**Table 3:**
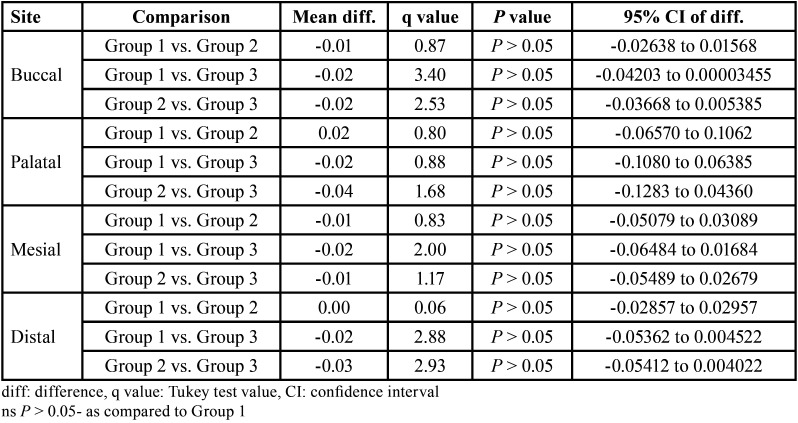
For each site, comparison (*P* value) of difference in pre to post mean change in dentin thickness (mm) between groups at 3 mm length by Tukey test.

II. At 5 mm length

The pre and post dentin thickness of three groups at 5 mm length in different sites (buccal, palatal, mesial and distal) is summarised in Table 4 for each site, comparing the difference in pre to post mean change in dentin thickness between groups (i.e. inter groups), Tukey test also showed significantly (*P* < 0.01 or *P* < 0.001) different and higher change in both Group 2 and Group 3 as compared to Group 1 at buccal site (Table 5 ). Moreover, it was also found significantly (*P* < 0.001) different and higher in Group 3 as compared to Group 1 at palatal site. However, at both, mesial and distal sites, it did not differ (*P* > 0.05) between groups i.e. found to be statistically the same.

**Table 4:**
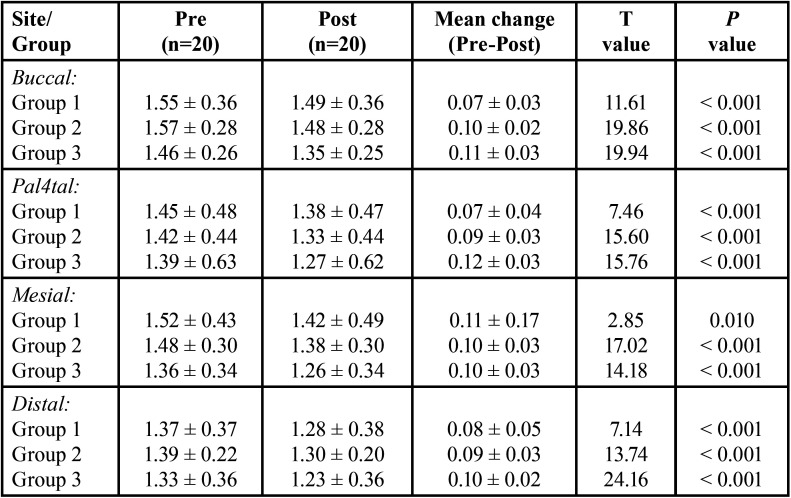
Pre and post dentine thickness (mm) of three groups at 5 mm length in different sites.

**Table 5:**
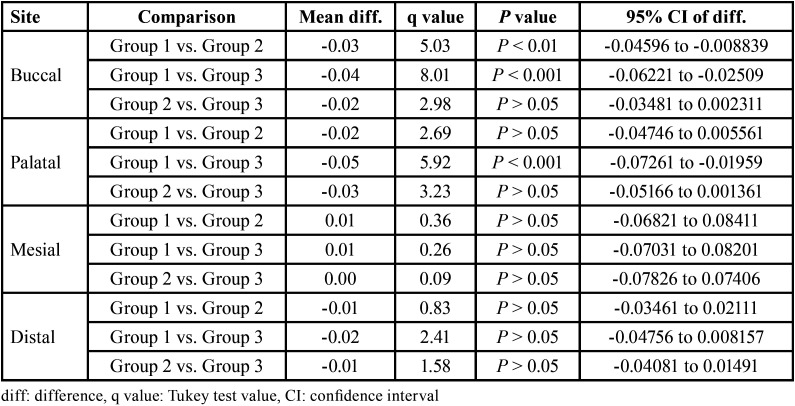
For each site, comparison (*P* value) of difference in pre to post mean change in dentin thickness (mm) between groups at 5 mm length by Tukey test.

III. At 7 mm length

The pre and post dentin thickness of three groups at 7 mm length in different ites (buccal, palatal, mesial and distal) is summarised in Table 6. Furthermore, for each site, comparing the difference in pre to post mean change in dentin thickness between groups (i.e. inter groups), Tukey test further showed similar (*P* > 0.05) pre to post mean change in dentin thickness between groups at buccal, mesial and palatal sites (Table 7). However, it palatal site, it was found significantly (*P* < 0.01) different and higher in Group 3 as compared to Group 1.

**Table 6:**
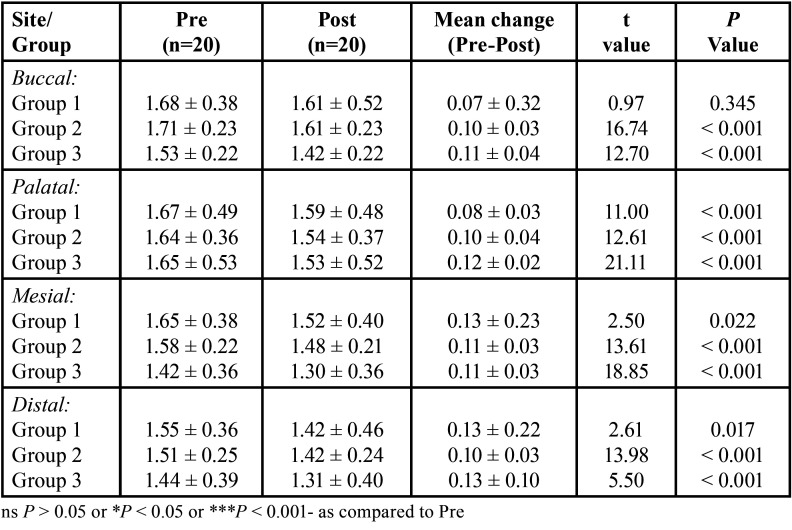
Pre and post dentine thickness (mm) of three groups at 7 mm length in different sites.

**Table 7:**
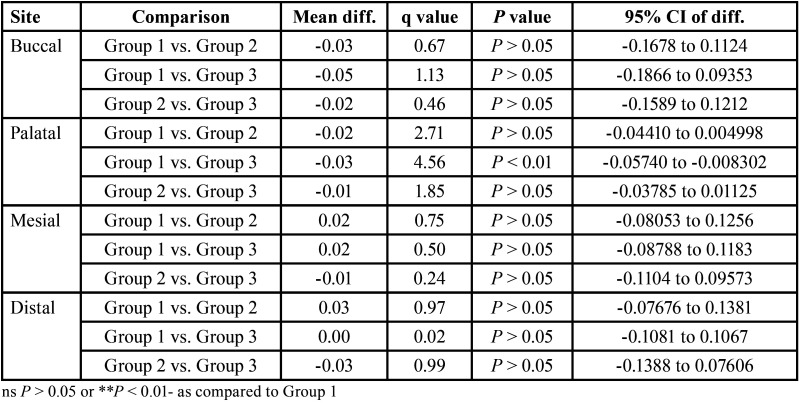
For each site, comparison (*P* value) of difference in pre to post mean change in dentin thickness (mm) between groups at 7 mm length by Tukey test.

## Discussion

Deciduous root canals are observed to be anatomically more tortuous & burdensome in comparison with the permanent teeth. Bacteria plays a vital role in the initiation, perpetuation of pulpal & periapical disease. One of the main objectives of endodontic therapy, is the elimination of micro-organisms from the root canals system which is achieved through removal of vital tissues, residual necrotic material, infected dentin & debris (8). Hand instrumentation, including the use of endodontic files & broaches, are now the bygone technique for treating the deciduous teeth & is time consuming as well (9). To overcome some of these issues, nickel-titanium alloy was introduced in endodontics which fulfilled the objectives of simplicity, speed, safety & stress reduction for both the patient & clinician (10). The quest for improvement lead to the development of better files system for making pulpectomy procedure successful, less time consuming which might have least or no error at all.

In this study, we have included an exclusive pediatric hand & rotary files designed for its use only in deciduous anterior teeth in which rotary files were used in rotary motion as well as reciprocation motion. Evaluation was done for dentine thickness at all the four walls i.e labial, palatal/lingual, mesial & distal walls & chairside time by counting the filing time for each canal preparation.

The results of our study showed lowered instrumentation time of both Group-2 & Group-3 as compared to Group 1. However, it did not differ (*P*>0.05) between Group-2 & Group-3 i.e found to be statistically same. Moreover, mean instrumentation time of Group-2 is lowered by 64.3% & 26.0% as compared to Group-1 & Group-3 respectively. Govindaraju L, *et al*. (11) assessed the instrumentation time with hand files, Pro Taper and Kedo-S rotary file in primary anterior teeth and concluded that pediatric file system decrease the instrumentation time which positively influences the cooperation of the children.

The results of our study showed that, the dentine thickness was measured in pre & post instrumentation from four walls (labial, palatal, mesial & distal). At 3mm the result showed that there is more dentine debridement in Reciprocating group in all the sites followed by rotary and hand filing group in facial, mesial and distal sites except for palatal site there is more dentine debridement in hand filing compared to rotary filing.

At 5mm, there is more dentine debridement in Reciprocating group followed by rotary and hand filing group in labial, palatal and distal sites except mesial site in which there is more debridement in hand filing group as compared to both rotary and hand filing group.

At 7 mm, there is more dentine removal in Reciprocating followed by Rotary and Hand filing group in labial and palatal sites. In mesial site there is more debridement in hand filing group as compared to rotary and hand filing group as there is equal dentine debridement. At distal site there is more and equal debridement in hand and rotary filing group as compared to reciprocating group.

The physics behind reciprocating motion is based on the law of action and reaction, which results in a balanced force during canal instrumentation as theoritized by Roane *et al*. (12). the reciprocating movement minimises the torsinal and flexural stresses, increases the canal centring ability and reduces the taper lock of the instrument within the canal and reducing the risk of root canal deformity. Unbalanced dentin removal was seen in rotary and hand file system, whereas reciprocating system showed more uniform removal of dentin, which is in accordance with our study. The rotary and reciprocating system showed more removal of dentin and more canal enlargement which is beneficial in pediatric patients because it helps the obturating material to flow easily inside the prepared canal space (13).

There are no studies in literature which have analysed the dentin thickness in deciduous teeth. In permanent teeth, Dhingra A, *et al*. (14) evaluated single file systems Rotary- One Shape and Reciprocating- Reciproc and Wave One on cervical dentin thickness using CBCT and they concluded that cervical dentin removal is maximum at all levels for One Shape and minimum for WaveOne showing better quality of preparation by Wave One and Reciproc over One Shape which is different from our study. Zinge PR *et al*. (15) also compared effect of rotary and reciprocating single-file systems on pericervical dentin in mandibular premolars and they concluded Reciprocating single-file System removes more pericervical dentin as compared to other rotary groups. In the present study also reciprocating files also showed more dentin removal, uniform and conical preparation.

In our results we found that Kedo- S square plus files have proven to be better in reciprocating motion in terms of dentinal microcracks and dentine thickness but takes slightly more time compared to rotary motion, which can be accepTable as there is no major time gap between these two motions.

## Conclusions

From the results of the present study following conclusions can be made:

1. Rotary filing takes least time for root canal preparation followed by reciprocating filing and the instrumentation time taken by hand filing was significantly higher.

2. Reciprocating filing helps in better dentin debridement at apical and the middle third and no difference was found at the coronal third among all three groups.
